# The Hidden Metabolic Roots of Epilepsy

**DOI:** 10.3390/biomedicines14040764

**Published:** 2026-03-27

**Authors:** Carmen Rubio, Sergio Carpinteyro, Norma Serrano-García, Héctor Romo-Parra, Javier Pérez-Villavicencio, Ángel Lee, Rodrigo Mercado-Pimentel, Moisés Rubio-Osornio

**Affiliations:** 1 Department of Neurophysiology, National Institute of Neurology and Neurosurgery, Mexico City 14269, Mexico; 2 Faculty of Medicine, Meritorious Autonomous University of Puebla, Puebla City 72000, Mexico; 3 Psychology Department, Ibero-American University, Santa Fe Campus, Mexico City 01376, Mexico; 4 Department of Electrical Engineering, Basic Sciences and Engineering Division, Metropolitan Autonomous University, Iztapalapa Campus, Mexico City 09340, Mexico; 5 National Institute of Public Health, Cuernavaca 62100, Mexico; 6 Hospital San Javier, Guadalajara 44620, Mexico; 7 Department of Neurochemistry, National Institute of Neurology and Neurosurgery, Mexico City 14269, Mexico

**Keywords:** epilepsy, metabolic syndrome, neuroinflammation, insulin resistance, gut-brain axis, mitochondrial dysfunction

## Abstract

The relationship between epilepsy, obesity, and metabolic syndrome (MetS) has emerged as a rapidly evolving area of neurobiology inquiry. Emerging evidence suggests that epilepsy extends beyond neuronal hyperexcitability, reframing it as a systemic condition characterized by significant metabolic dysregulation. Converging supports a bidirectional relationship while seizures, antiseizure medications (ASM), and neuroinflammation induce exacerbate potentiate epileptogenesis through shared molecular pathways. At the cellular level, chronic epileptic activity induces oxidative stress, mitochondrial dysfunction, and the activation of microglia and astrocytes. This, in turn, leads to the release of pro-inflammatory cytokines including TNF-α, IL-1β, and IL-6. These mediators traverse the blood-brain barrier (BBB), subsequently modifying insulin signaling, and disrupting glucose homeostasis, which collectively fosters a pro-inflammatory and insulin-resistant environment. Furthermore, antiseizure medications such as valproate can exacerbate these effects by directly impairing insulin receptor signaling and altering adipokine production, ultimately contributing to weight gain and systemic metabolic dysregulation. Obesity and MetS induce neuroinflammatory and excitotoxic states that promote seizure onset via leptin resistance, reduced adiponectin levels, and compromised AMP-activated protein kinase (AMPK) signaling. Emerging evidence emphasizes the gut-brain axis as a crucial regulator in this reciprocal interaction. Dysbiosis, altered microbial metabolites (e.g., short-chain fatty acids), and heightened intestinal permeability facilitate systemic inflammation and BBB disruption, enhancing neuronal excitability. Insulin resistance in the brain disrupts synaptic transmission, impairs mitochondrial biogenesis, and compromises redox equilibrium, perpetuating a pathological cycle linking metabolic stress to epileptic activity. This review synthesizes the cellular, molecular, and systemic pathways connecting epilepsy, obesity, and MetS, and proposes that epilepsy be reconceptualized as a neuro-metabolic disorder. Insights into these convergent pathways provide a rationale for novel therapeutic strategies that simultaneously target seizure control and metabolic regulation, encompassing microbiota modulation, antioxidant therapy, and insulin-sensitizing interventions with the overarching aim of restoring neuro-metabolic homeostasis.

## 1. Introduction

Epilepsy is a global neurological disorder affecting approximately 70 million people and contributing significantly to the burden of disability-adjusted life years (DALYs) across all age groups [1]. While traditionally defined by recurrent, unprovoked seizures, epilepsy is increasingly recognized as a systemic condition that impacts not only the central nervous system (CNS) but also involves intricate metabolic, immunological, and endocrine alterations [2]. Recent data revealed a troubling association between epilepsy and metabolic illnesses, such as obesity and MetS, which have reached pandemic proportions globally [3]. This intersection of neurological and metabolic dysfunction represents a complex and reciprocal connection where epilepsy and metabolic alterations mutually exacerbate one another through shared cellular and molecular pathways [4]. Consequently, the realization that metabolic disturbances can modulate neuronal excitability and seizure thresholds has fundamentally shifted our understanding of epilepsy’s etiology [5]. Historically, obesity and MetS were viewed as secondary comorbidities resulting from ASM therapy or lifestyle restrictions [6]. However, emerging evidence suggests these metabolic irregularities may contribute to both epileptogenesis and seizure progression [7]. This paradigm shift underscores the necessity of framing epilepsy not merely as an isolated brain disorder, but as a multifaceted systemic syndrome driven by chronic inflammation, oxidative stress, adipokine dysregulation, and insulin resistance [8]. The metabolic profile of individuals with epilepsy is a crucial determinant of disease progression, treatment effectiveness, and overall prognosis [9]. Epidemiological studies consistently demonstrate that persons with epilepsy exhibit a much higher prevalence of overweight, obesity, and metabolic syndrome relative to the general population [10].

Numerous comprehensive meta-analyses have shown that 40–60% of adults with epilepsy meet the criteria for metabolic syndrome, with obesity rates significantly exceeding those of matched healthy controls [11]. Importantly, this association remained notable even when considering ASM usage, suggesting the involvement of inherent disease-related mechanisms [12].

Obesity and MetS, through systemic inflammation, endothelial dysfunction, and hormonal alterations, appear to increase neuronal excitability and lower the seizure threshold, establishing a self-perpetuating cycle of neuro-metabolic dysfunction [13]. This bidirectional relationship creates a novel pathogenic continuity between the brain and peripheral metabolism, contesting conventional neurocentric models of epilepsy [14]. Epilepsy and obesity share essential biological traits, including mitochondrial dysfunction, redox imbalance, and the activation of pro-inflammatory pathways [15,16]. In epilepsy, recurrent seizures trigger the activation of microglia and astrocytes, leading to the secretion of cytokines such as interleukin-1β (IL-1β), tumor necrosis factor-α (TNF-α), and interleukin-6 (IL-6) [17]. These mediators not only modulate neuronal excitability within epileptogenic circuits but also enter systemic circulation, thereby impairing peripheral insulin signaling and dysregulating lipid metabolism [4]. Conversely, visceral adipose tissue in obesity functions as a metabolically active endocrine organ that continuously secretes pro-inflammatory adipokines and cytokines [18,19]. Extended exposure to these circulating mediators might undermine the integrity of the BBB, facilitating neuroinflammatory cascades that heighten the susceptibility of neuronal networks to hyperexcitability [20]. As a result, a reciprocal feedback loop emerges in which central neuroinflammation and peripheral metabolic irregularities mutually reinforce each other across multiple biological levels [21].

A crucial element of this bidirectional model is the effect of ASM, particularly valproate (VPA), carbamazepine, and gabapentin, which substantially affect energy balance and endocrine function [6,22]. Valproate (VPA), a commonly prescribed broad-spectrum antiseizure drug, is strongly associated with weight gain, insulin resistance, and dyslipidemia [22,23]. At the molecular level, VPA impairs insulin signaling by altering the phosphorylation of insulin receptor substrates (IRS-1/2) and inhibiting GLUT4 translocation in adipocytes and skeletal muscle [24]. These alterations lead to reduced glucose uptake, compensatory hyperinsulinemia, and systemic insulin resistance key features of MetS [23]. Moreover, VPA modulates adipokine secretion by adiponectin levels and increasing leptin concentrations, thereby promoting leptin resistance and enhancing appetite and energy storage [25]. The metabolic side effects are particularly detrimental in individuals with epilepsy, as they can elevate seizure recurrence, exacerbate cognitive impairment, and increase cardiovascular risk [2]. Besides pharmaceutical factors, seizures may induce systemic alterations that elevate the risk of metabolic imbalance [4]. Recurrent neuronal hyperactivity increases the metabolic demands of neurons and glia, leading to mitochondrial stress and excessive generation of reactive oxygen species (ROS) [15,26]. Oxidative stress damages brain structures and affects peripheral tissues, such as the liver and muscle, disrupting mitochondrial β-oxidation and promoting fat accumulation [27]. The convergence of mitochondrial dysfunction and oxidative stress establishes a shared causal pathway linking epilepsy and metabolic disorders [28]. Furthermore, chronic endoplasmic reticulum stress caused by glucose dysregulation and the accumulation of ROS can trigger the unfolded protein response (UPR), impede protein folding, and promote neuronal death, thereby worsening metabolic dysfunction and epileptogenesis [29,30]. A vital component of this neuro-metabolic axis is adipokine dysregulation, linking peripheral metabolic signals to central neuronal activity [31]. Adipokines, such as leptin, adiponectin, and resistin, directly affect synaptic transmission and plasticity [4,32]. Under physiological conditions, leptin modulates GABAergic and glutamatergic signaling, exerting anticonvulsant and neuroprotective effects [33,34]. In obesity, leptin resistance diminishes these advantages and results in hyperexcitability [35]. Similarly, reduced adiponectin levels commonly observed in obese and insulin-resistant patients impede the activation of the AMPK pathway, hence diminishing neuronal resistance to oxidative and metabolic stress [36]. These hormonal alterations collectively create a metabolic milieu that increases neuronal vulnerability, irregular excitability, and the initiation of seizures [13]. Insulin resistance in the CNS is a significant and compelling aspect of this emerging field [37]. Insulin-independent glucose transporters are mostly found in the brain; nonetheless, insulin signaling is essential for neuronal survival, synaptic plasticity, and neurotransmitter regulation [38,39].

Cerebral insulin resistance hinders these functions, diminishing inhibitory GABAergic activity and augmenting excitatory glutamatergic transmission, hence enhancing seizure susceptibility [40]. Furthermore, impaired insulin signaling exacerbates oxidative stress and mitochondrial dysfunction, both of which are recognized factors in epileptogenesis [41]. The concept of “type 3 diabetes” in the brain, previously examined concerning Alzheimer’s disease, may also pertain to epilepsy, providing a unified framework for understanding how systemic metabolic dysfunction results in neuronal instability [42]. Understanding the bidirectional relationship between epilepsy and metabolic illnesses has considerable therapeutic implications from a translational standpoint [2]. Modern therapeutic strategies for PWE rarely include metabolic assessment or interventions beyond seizure control [43]. Understanding that metabolic dysregulation significantly influences epileptogenesis presents new avenues for targeted therapy [7,44]. Nutritional modifications, anti-inflammatory medicines, and insulin-sensitizing drugs may augment conventional antiseizure treatments, addressing both neurological and metabolic dimensions of the illness [4,45].

Additionally, identifying indicators of metabolic stress in PWE may facilitate the early identification of comorbidities and the formulation of personalized treatment strategies [43]. This bidirectional network improves our understanding of epileptogenesis and underscores the importance of systemic metabolic health in neurological resilience [2]. Elucidating the cellular and molecular linkages between the brain and peripheral metabolism offers a promising pathway for the development of integrative therapeutic strategies that transcend the traditional boundaries of neurology and endocrinology [5].

This study posits that epilepsy and metabolic syndrome are interconnected through shared cellular and molecular mechanisms, specifically neuroinflammation, oxidative stress, adipokine dysregulation, and insulin resistance, which establish a bidirectional pathogenic loop that exacerbates both neuronal hyperexcitability and systemic metabolic dysfunction [4]. Consequently, this research aims to analyze and integrate existing evidence regarding the bidirectional relationship between epilepsy, obesity, and metabolic syndrome. By emphasizing the mechanisms linking these conditions, the study seeks to identify potential therapeutic targets for integrated neuro-metabolic intervention.

## 2. Chronic Inflammation and Oxidative Stress

**Figure 1:**
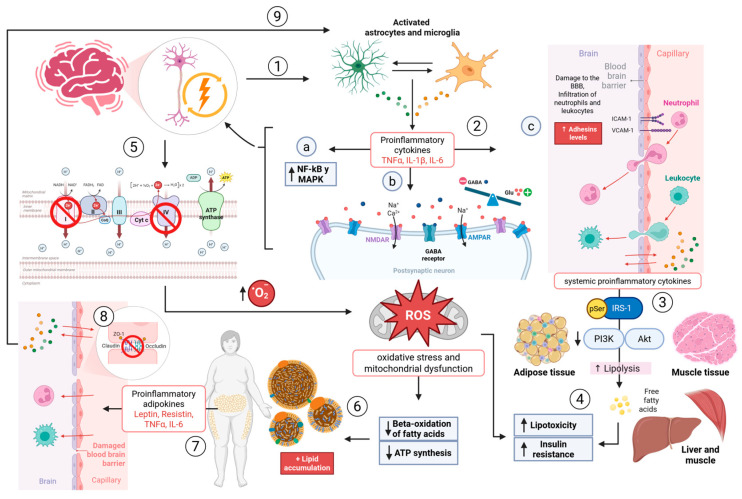
**Neuroinflammation cycle. 1.** Recurrent epileptic activity stimulates microglia and astrocytes to release pro-inflammatory cytokines IL-1b, TNF-a, IL-6. **2a.** Cytokines exacerbate neuroinflammation by activating the NF-kB and MAPK pathways. **2b.** Cytokines modify neuronal excitability by upregulating NMDA receptors (↑), α-amino-3-hydroxy-5-methyl-4-isoxazolepropionic acid (AMPA) receptors (↑), and GABA-A receptors (↓). They also affect the functionality of ion transporters: K_ATP_ and Na/K ATPase, (↑ membrane depolarization ↑ epileptic seizures). **2c.** Cytokines alter the BBB by increasing the expression of ICAM-1, VCAM-1; allowing infiltration of peripheral immune cells into the CNS. **3.** Cytokines released from the CNS affect peripheral metabolism, such as in adipose and muscle tissue, by facilitating serine phosphorylation of IRS-1, disrupting the PI3K-AKT pathway, leading to lipolysis and the release of free fatty acids. **4.** FFA lead to lipotoxicity and insulin resistance in the liver and muscle, due to mitochondrial stress and increased ROS. **5.** Chronic epileptic activity leads to increased oxidative stress and mitochondrial dysfunction in neurons and glial cells, due to decreased activity in the electron transport chain and generation of superoxide anions. **6.** Mitochondrial insufficiency alters the beta-oxidation of fatty acids and ATP synthesis in the liver and muscle, leading to lipid accumulation. **7.** In obesity, adipose tissue secretes pro-inflammatory adipokines: leptin, resistin, TNF-α, IL-6, which perpetuate chronic inflammation. **8.** IL-6 and TNF-α compromise the BBB by decreasing the expression of occludin, claudin-5, and ZO-1, facilitating the entry of cytokines and peripheral immune cells into the CNS. **9.** Microglia activated by the passage of pro-inflammatory molecules perpetuate the neuroinflammation cycle. Upward arrows (↑) indicate increased expression, activity, or upregulation; downward arrows (↓) indicate decreased expression, activity, or downregulation. Blunt-ended lines (⊣) represent inhibitory signals. Numbered arrows (1 to 9) indicate the sequential steps of the neuroinflammation cycle described above. Colored circles represent different molecular species: green circles, glutamate (Glu); pink/red circles, pro-inflammatory cytokines and adipokines; blue circles, GABA; yellow/orange circles, reactive oxygen species (ROS) and lipid-related molecules. The double-headed arrows (↔) between microglia and astrocytes indicate bidirectional activation. Created in BioRender. Carpinteyro, S. (2026) https://BioRender.com/ie3qud3 ( accessed on 23 March 2026).

Epilepsy constitutes a persistent pro-inflammatory condition. The neuroinflammatory response in epilepsy encompasses a broad repertoire of molecular mediators, including pro-inflammatory cytokines, chemokines (such as CCL2, CXCL10), complement proteins, prostaglandins, and matrix metalloproteinases, all of which contribute to glial activation, BBB disruption, and neuronal hyperexcitability [46]. The present review focuses on interleukin-1β (IL-1β), tumor necrosis factor-α (TNF-α), and interleukin-6 (IL-6) as the primary illustrative mediators of this cascade, because: (i) they are among the most consistently elevated cytokines in both human epilepsy tissue and animal seizure models; (ii) they have established direct effects on glutamatergic and GABAergic receptor function; and (iii) their downstream signaling via the nuclear factor kappa B (NF-κB), mitogen-activated protein kinase (MAPK), and the Janus kinase/signal transducer and activator of transcription (JAK/STAT) pathways integrates neuroinflammation with the peripheral metabolic dysfunction that is the central focus of this review. This focus is not intended to minimize the contribution of other mediators, but to provide a mechanistically coherent framework for the neuro-metabolic model proposed here. Recurrent epileptic seizures stimulate microglia and astrocytes triggering the release of a broad array of pro-inflammatory mediators most notably interleukin-1 beta (IL-1β), tumor necrosis factor alpha (TNF-α), and interleukin-6 (IL-6), among others which exacerbate neuroinflammation and disrupt neuronal homeostasis through the activation of NF-κB and MAPK signaling pathways [17,46] (Figure 1). These cytokines extend beyond the CNS; they can traverse the BBB and affect peripheral metabolism by modifying endothelial permeability and facilitating systemic inflammatory responses [8,47]. As a preclinical evidence, TNF-α potently inhibits insulin signaling in adipose and muscle tissues by promoting the serine phosphorylation of IRS-1. This disruption of the PI3K-AKT pathway triggers lipolysis and the subsequent release of free fatty acids (FFA) [48,49]. The elevation of circulating FFA leads to lipotoxicity and insulin resistance in the liver and muscle due to mitochondrial stress and increased ROS production [50,51]. Chronic epileptic activity correlates with heightened oxidative stress and mitochondrial malfunction in neurons and glial cells, characterized by diminished activity of complexes I and IV of the electron transport chain and excessive generation of superoxide anions [15,26]. Mitochondrial dysfunction may affect metabolically active tissues, including the liver and muscle, impairing fatty acid β-oxidation and ATP synthesis, hence leading to lipid buildup and systemic metabolic dysfunction [27]. Clinical Evidence show that the obesity and MetS are not merely outcomes of epilepsy; they can also serve as independent risk factors for the onset and progression of the condition by promoting systemic inflammation, hyperglycemia, and oxidative stress that lower the seizure threshold [4]. Systemic inflammation and insulin resistance associated with MetS establish a neuronal milieu conducive to epileptogenesis [14]. In obesity, visceral adipose tissue functions as an active endocrine organ, secreting large amounts of pro-inflammatory adipokines, including leptin, resistin, and TNF-α, which sustain chronic low-grade inflammation [18,19]. Chronic systemic inflammation, marked by elevated IL-6 and TNF-α levels, might undermine the integrity of the BBB by downregulating tight-junction proteins including occludin and claudin-5 [20]. A weakened BBB facilitates the entry of peripheral immune cells and cytokines into the CNS, activating resident microglia and sustaining a cycle of neuroinflammation [21]. Neuroinflammation is a primary factor in epileptogenesis, as cytokines modify neuronal excitability and synaptic plasticity by regulating N-methyl-D-aspartate (NMDA) receptor activation and glutamate release [13,52]. Chronic inflammation can impair the functionality of ionic transporters and potassium channels, including ATP-sensitive K^+^ (K_ATP_) channels and Na^+^/K^+^-ATPase, which are essential for sustaining neuronal membrane potential and averting hyperexcitability [53].

## 3. Insulin Resistance and Neuronal Impairment (Preclinical Evidence)

**Figure 2:**
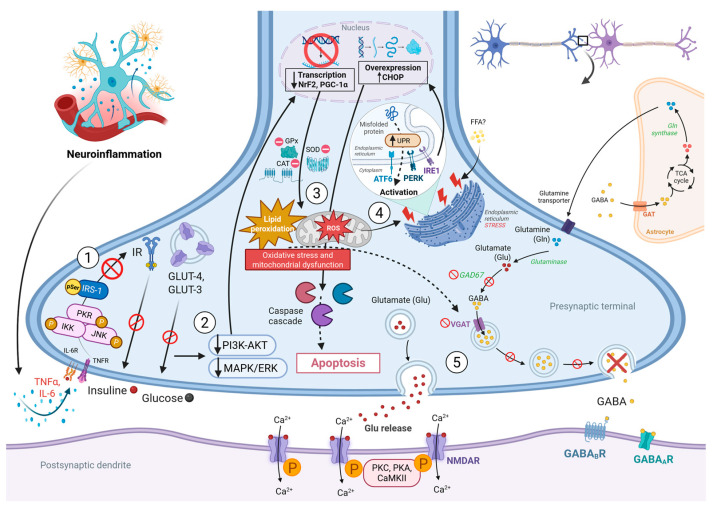
**Neuronal deterioration due to cerebral insulin resistance. 1.** Under conditions of insulin resistance and neuroinflammation, TNF-α and IL-6 facilitate serine phosphorylation and IRS-1 inactivation, with decreased IR expression and consequent reduction in GLUT3 and GLUT4 translocation in neurons and astrocytes. **2.** Reduced sensitivity to IR activation results in decreased intracellular signaling of neurons, astrocytes, and microglia, affecting PI3K-AKT and MAPK/ERK pathways, causing insulin to lose its neuromodulatory functions. **3.** Through dysfunction of the PI3K-AKT pathway, Nrf2 and PGC-1a transcriptional activity is decreased, leading to lower production of antioxidant enzymes (Superoxide dismutase (SOD), catalase (CAT) and glutathione peroxidase (GPx), consequently the accumulation of ROS and lipid peroxidation byproducts, thus inducing oxidative stress and mitochondrial dysfunction. **4.** The metabolic overload associated with insulin resistance causes ER stress due to the accumulation of ROS and misfolded and unfolded proteins, activating the UPR through the PERK, IRE1, and ATF6 pathways. This chronic UPR activation results in overexpression of C/EBP homologous protein (CHOP), inducing mitochondrial dysfunction and caspase-dependent cell death. **5.** Cerebral insulin resistance modifies neurotransmission, decreasing inhibitory GABAergic action due to lower expression of GAD67 and vesicular GABA transporters, while increasing excitatory glutamatergic transmission with greater phosphorylation of NMDA receptors. Upward arrows (↑) indicate increased expression, activity, or upregulation, whereas downward arrows (↓) indicate decreased expression, activity, or downregulation. The black box delineates the dendritic region and its associated synapses. Colored circles represent the release of different molecules, such as IL-6 and GABA.Created in BioRender. Carpinteyro, S. (2026) https://BioRender.com/ugvy52c (accessed on 23 March 2026).

As an energy-intensive organ, the brain relies primarily on glucose metabolism Consequently, insulin resistance significantly disrupts neuronal homeostasis by restricting glucose uptake in neurons via the downregulation of insulin-sensitive glucose transporters, such as GLUT4 and GLUT3, in neuronal and astrocytic membranes [37,54] (Figure 2). Beyond its metabolic role, insulin functions as a neuromodulator at the cellular level by binding to insulin receptors (IR) found in hippocampal and cortical neurons. This binding initiates downstream PI3K-Akt and MAPK signaling pathways that regulate gene transcription, synaptic plasticity, and neuronal survival [38,39]. Cerebral insulin resistance modifies neurotransmission by diminishing inhibitory GABAergic signaling due to decreased expression of glutamic acid decarboxylase 67 (GAD67) and vesicular γ-Aminobutyric Acid (GABA) transporters, while concurrently augmenting excitatory glutamatergic transmission through increased phosphorylation of NMDA receptors, thereby shifting the excitatory/inhibitory equilibrium towards neuronal hyperexcitability and seizure onset [40,55].

Metabolic overload linked to insulin resistance causes endoplasmic reticulum (ER) stress in neurons and astrocytes due to the buildup of misfolded and unfolded proteins, thereby activating the UPR through the PERK, IRE1, and ATF6 signaling pathways [29,30]. Chronic activation of the UPR results in the overexpression of C/EBP homologous protein (CHOP), which induces mitochondrial malfunction and caspase-dependent death, especially in inhibitory interneurons essential for neural network integrity [56,57,58]. Obesity and MetS exacerbate these cellular disruptions by increasing systemic concentrations of pro-inflammatory cytokines, including TNF-α, IL-6, and leptin, which traverse the BBB and hinder insulin receptor signaling via serine phosphorylation and the inactivation of IRS-1 in neurons and astrocytes [41,59]. The inflammation-induced insulin resistance exacerbates mitochondrial oxidative stress, resulting in the overproduction of ROS, lipid peroxidation, and oxidative damage to neuronal membranes, mitochondrial DNA, and synaptic proteins associated with vesicular trafficking and neurotransmitter release [28,60]. In clinical and translational evidence, the association between epilepsy and metabolic syndrome constitutes a complex bidirectional feedback loop wherein recurrent seizures induce systemic inflammation and insulin resistance, while obesity and metabolic syndrome intensify neuroinflammation, oxidative stress, and synaptic dysfunction, thereby creating a detrimental cycle that heightens seizure susceptibility and accelerates cognitive decline [2,4,7,11]. Comprehending these interrelated molecular pathways is essential for formulating treatment strategies to restore insulin signaling, mitigate ER stress, and diminish neuronal hyperexcitability in individuals with epilepsy [61,62].

## 4. Adipose Tissue as an Endocrine Organ: Adipokine Dysregulation and Neuroinflammation in Epilepsy

**Figure 3:**
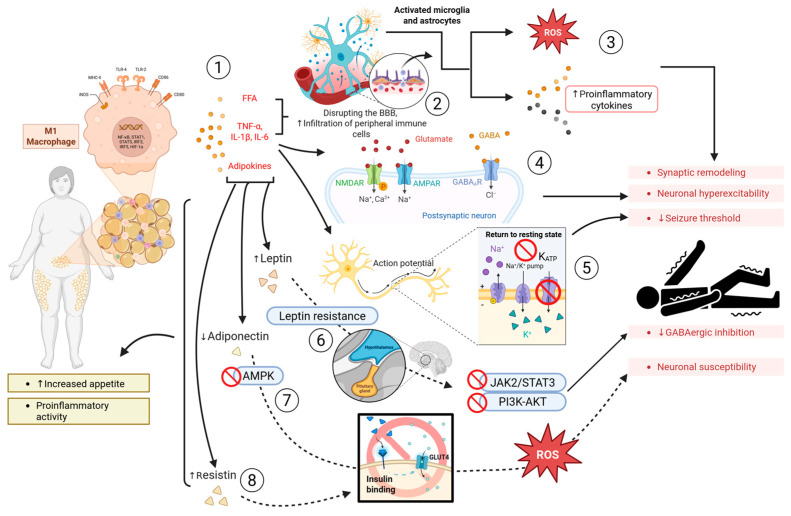
**Obesity-epilepsy. 1.** In obesity, visceral adipose tissue acts as an active endocrine organ; hypertrophic adipocytes and infiltrating macrophages release pro-inflammatory cytokines: TNF-α, IL-6, IL-1β; adipokines and FFA. **2.** Released cytokines and FFA can cross and disrupt the BBB, promoting infiltration of peripheral immune cells into the central nervous system. **3.** Recruited immune cells, along with activated microglia and astrocytes, secrete ROS and other cytokines that facilitate neuronal hyperexcitability and synaptic remodeling. **4.** Cytokines directly affect neuronal signaling by increasing surface expression of AMPA glutamate receptors, amplifying NMDA receptor phosphorylation, and simultaneously reducing GABA-A receptor activity. **5.** Prolonged exposure to cytokines modifies activation characteristics of ion channels, especially ATP-sensitive potassium (K_ATP_) channels; blockade of K_ATP_ channels causes membrane depolarization, lowering the seizure threshold. **6.** Obesity leads to dysregulation of adipokines; hyperleptinemia accompanied by central leptin insensitivity (leptin resistance) alters hypothalamic signaling by affecting the JAK2/STAT3 and PI3K-AKT pathways, resulting in increased appetite and decreased GABAergic inhibition. **7.** The decrease in adiponectin levels in obesity hinders the activation of the AMPK pathway, causing it to lose its insulin-sensitizing and anti-inflammatory functions, thus intensifying oxidative stress and neuronal susceptibility. **8.** Resistin is released by adipose tissue in obesity, promoting insulin resistance and the release of pro-inflammatory cytokines. Upward arrows (↑) indicate increased expression, activity, or upregulation; downward arrows (↓) indicate decreased expression, activity, or downregulation. Created in BioRender. Carpinteyro, S. (2026) https://BioRender.com/nh783nu (accessed on 23 March 2026).

Obesity should be regarded not just as an accumulation of adipose tissue, but as a chronic condition of metabolic and endocrine dysfunction that significantly disrupts systemic and neural homeostasis via the release of bioactive substances known as adipokines and pro-inflammatory cytokines [16,32,63,64]. This pathophysiological situation creates a reciprocal relationship between obesity, MetS, and epilepsy, in which systemic metabolic dysregulation and neuroinflammation mutually exacerbate neuronal excitability and increase seizure vulnerability [65,66,67]. The primary processes driving this association include neuroinflammatory activation, adipokine dysregulation, and insulin resistance, which intersect at shared molecular targets like mitochondria, glial cells, and synaptic ion channels [68]. In obesity, preclinical evidence visceral adipose tissue converts into an active endocrine organ characterized by hypertrophic adipocytes and infiltrating macrophages that release elevated levels of pro-inflammatory mediators such as TNF-α, IL-6, and IL-1β, resulting in a condition of chronic low-grade inflammation [18,19,69] (Figure 3). This situation is sustained by macrophage polarization towards the M1 phenotype, which enhances cytokine release and oxidative stress via NF-κB and JNK signaling pathways [70,71]. Cytokines and free fatty acids released from adipose tissue can traverse or communicate over the BBB, provoking neuroinflammatory reactions in the CNS [72,73]. Cytokines like IL-1β and TNF-α increase the expression of ICAM-1 and VCAM-1 in BBB endothelial cells, hence promoting leukocyte adherence and the infiltration of peripheral immune cells into the brain parenchyma [74,75,76]. The recruited immune cells, along with activated microglia and astrocytes, secrete ROS, nitric oxide, and other cytokines that facilitate neuronal hyperexcitability and synaptic remodeling [77,78]. TNF-α and IL-1β directly affect neuronal signaling by altering neurotransmitter receptor functionality. TNF-α increases the surface expression of AMPA-type glutamate receptors, while IL-1β amplifies NMDA receptor phosphorylation and concurrently reduces GABA-A receptor-mediated inhibitory currents, thereby altering the synaptic equilibrium in favor of excitation [79,80]. Moreover, prolonged exposure to TNF-α facilitates the degradation of tight junction proteins, including occludin, claudin-5, and ZO-1, in BBB endothelial cells, resulting in enhanced barrier permeability and permitting the ingress of proconvulsant molecules, such as albumin and peripheral cytokines, into the brain parenchyma [72,74,81]. The introduction of these chemicals sustains microglial activation and astrocytic gliosis, perpetuating a self-reinforcing loop of inflammation and neuronal hyperexcitability [77]. Extended exposure to cytokines modifies the expression and gating characteristics of voltage-gated Na^+^, Ca^2+^, and K^+^ channels, especially the K_ATP_ channels, which govern neuronal membrane potential and excitability thresholds [53,79,82]. The blockage of K_ATP_ channels by cytokines leads to membrane depolarization and increased neuronal firing, facilitating seizure development [80,82]. Furthermore, preclinical and clinical evidence show that the adipokines peptide hormones secreted by adipose tissue, including leptin, adiponectin, and resisting are essential in energy metabolism and synaptic function [83,84,85]. Obesity leads to an adipokine imbalance, characterized by elevated leptin and reduced adiponectin levels, which disrupts hypothalamic and hippocampal signaling. By modulating the AMPK, JAK/STAT, and PI3K-Akt pathways, this imbalance impacts neuronal glucose utilization and excitability [86,87]. Notably, the relationship between leptin and seizure susceptibility remains contentious. While experimental rodent models consistently report the anticonvulsant effects of leptin via Ob-R/JAK2-STAT3 signaling evidenced by reduced seizure duration in kainic acid models cross-sectional clinical studies of patients with epilepsy report elevated serum leptin levels that do not correlate with a reduction in seizure frequency [88]. This apparent paradox likely reflects central leptin resistance, in which receptor downregulation at the hypothalamic level uncouples circulating leptin from its neuroprotective function. Similarly, the directionality of adiponectin’s effect on neuronal excitability requires further validation in prospective human cohorts, as most mechanistic data derive from rodent knockout models. This adipokines’ dysregulation, coupled with persistent inflammation, establishes a molecular axis that connects metabolic dysfunction, neuroinflammation, and epileptogenesis. The cellular and molecular roles of the principal adipokines involved in this axis are summarized in Table 1.

## 5. A Molecular Link Among Obesity, Metabolic Syndrome, and Epilepsy in Cerebral Insulin Resistance

Building on the insulin signaling framework described in Section 3, cerebral insulin resistance constitutes the molecular bridge through which obesity and MetS directly impair neuronal function. Beyond its peripheral manifestations, this resistance state converges with adipokine dysfunction to create a self-amplifying neuro-metabolic loop [63]. Recent evidence supports the existence of cerebral insulin resistance, characterized by diminished sensitivity of neurons, astrocytes, and microglia to insulin receptor (IR) activation, resulting in altered intracellular signaling and disrupted energy balance [54,94,95]. In the brain, insulin mediates critical crucial neuroprotective and neuromodulatory functions by activating the PI3K, Akt, and MAPK/ERK pathways to regulate neuronal survival, synaptic plasticity, and neurotransmitter receptor expression [96,97]. Under physiological conditions, insulin signaling facilitates glucose uptake through GLUT4 and GLUT3 transporters expressed on neuronal and astrocytic membranes, thereby maintaining adequate energy supply for synaptic activity and ion homeostasis [94,98]. In insulin-resistant conditions, serine phosphorylation of IRS-1 and the downregulation of IR expression disrupt these signaling pathways, diminishing GLUT translocation and leading to neuronal glucose hypometabolism, a characteristic noted in epileptogenic brain regions [96,99,100] (Figure 2).

Preclinical evidence shows that insulin acts as a modulator of excitatory and inhibitory neurotransmission at the synaptic level. It enhances GABAergic signaling by preserving the expression of GAD67 and vesicular GABA transporters, while concurrently inhibiting glutamatergic activity through the modulation of NMDA receptor phosphorylation [99,101]. Insulin signaling failure results in diminished inhibitory tone and heightened excitatory drive, causing synaptic imbalance, neuronal hyperexcitability, and increased seizure susceptibility [96]. Cerebral insulin resistance at the molecular level induces oxidative stress and mitochondrial dysfunction, both of which are pivotal in epileptogenesis [90,102]. The malfunctioning PI3K and Akt signaling diminishes the transcriptional activity of Nrf2 and PGC-1α, leading to decreased production of antioxidant enzymes such as SOD, catalase, and glutathione peroxidase, while also hindering mitochondrial biogenesis [94,103]. Consequently, there is an accumulation of ROS and lipid peroxidation byproducts, which undermine mitochondrial membrane potential, ATP synthesis, and neuronal survival [90,104]. As a translational perspective, insulin resistance into the CNS, neuroinflammation, and adipokine dysfunction (Table 1) serve as molecular links connecting obesity, metabolic syndrome, and epilepsy, creating a self-perpetuating cycle that maintains both metabolic and neurological dysfunction [65,68,105]. Despite the compelling mechanistic evidence linking cerebral insulin resistance to epileptogenesis, important limitations must be acknowledged. Most data on IRS-1 serine phosphorylation, GLUT downregulation, and GAD67 suppression in epileptic conditions derive from animal models, and direct evidence of these specific molecular changes in human epileptic brain tissue remains scarce. Furthermore, whether cerebral insulin resistance is a primary driver of seizure initiation or a consequence of recurrent seizure activity —and the associated metabolic demand— has not been definitively resolved in clinical studies. Prospective longitudinal cohorts with metabolic biomarker profiling are needed to establish temporal directionality. A deeper understanding of these convergent pathways may inform therapeutic strategies targeting insulin sensitization, redox regulation, and neuroinflammatory modulation, with the potential to improve seizure outcomes and metabolic homeostasis in individuals with epilepsy [62,63].

## 6. The Gut–Brain Axis in the Epilepsy–Obesity Connection: Cellular and Molecular Mechanisms

**Figure 4:**
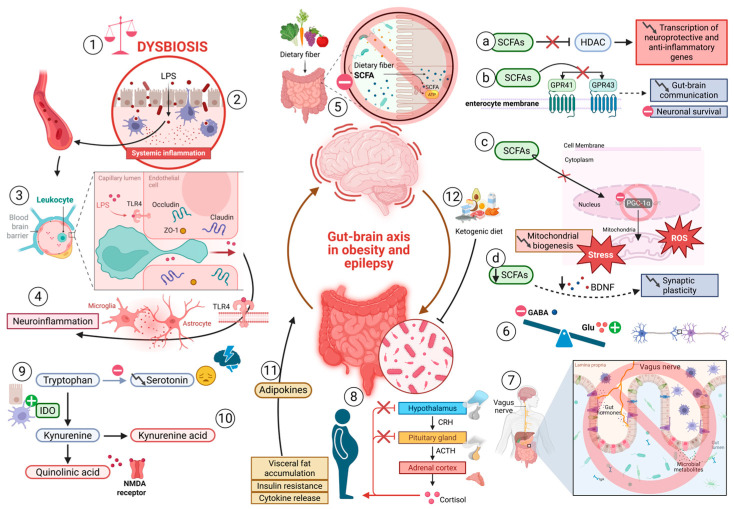
**Gut-brain axis as a connection between epilepsy and obesity. 1.** In epilepsy, intestinal dysbiosis is present, with lower microbial diversity of beneficial genera and a higher prevalence of pro-inflammatory taxa. **2.** Microbial dysbiosis induces systemic inflammation and compromises the intestinal barrier, allowing bacterial endotoxins such as LPS to enter the bloodstream. **3.** Circulating LPS activates TLR4 receptor signaling in endothelial cells (BBB), leading to a decrease in tight junction proteins, which facilitates infiltration of immune cells into the CNS. **4.** Activation of TLR4 receptor signaling by LPS in microglia and astrocytes leads to the synthesis of pro-inflammatory cytokines, perpetuating neuroinflammation. **5.** In obesity and epilepsy, the synthesis of SCFA such as butyrate, propionate, and acetate is significantly reduced, leading to: **a.** Reduction in its inhibitory function of histone deacetylases (HDAC), which decreases the transcription of neuroprotective and anti-inflammatory genes. **b.** Reduced activation of GPR41 and GPR43 receptors in enterocytes, preventing key signaling pathways in gut-brain communication, and consequently reducing neuronal survival. **c.** The altered signaling of SCFA decreases the expression of PGC-1α, interfering with mitochondrial biogenesis and oxidative metabolism by decreasing oxidative phosphorylation and increasing ROS production, ultimately perpetuating oxidative stress. **d.** Furthermore, the decrease in microbial metabolites leads to lower brain-derived neurotrophic factor (BDNF) expression, resulting in reduced synaptic plasticity. **6.** Microbial dysbiosis promotes an imbalance in neurotransmitter production towards an excitatory predominance, with decreased GABA synthesis and increased glutamatergic activity **7.** In obesity and epilepsy, vagal tone is reduced, decreasing parasympathetic control and the cholinergic anti-inflammatory reflex. **8.** Dysautonomia increases activation of the HPA axis, leading to cortisol secretion. Chronic dysregulation of this axis exacerbates visceral fat accumulation, insulin resistance, and increased cytokine release. **9.** In microbial dysbiosis and inflammatory states, enterocytes and immune cells produce excess IDO, promoting the diversion of tryptophan metabolism from serotonin synthesis to kynurenine biosynthesis. **10.** The neuroactive metabolites quinolinic acid and kynurenic acid cross the blood-brain barrier and influence glutamatergic transmission. Quinoline acid acts as an NMDA receptor agonist. Decreased serotonin levels lead to depressive and cognitive symptoms. **11.** Adipokines (ghrelin, leptin, adiponectin) regulate the gut-brain axis (GBA). **12.** Ketogenic diet modulates the intestinal microbiota. Created in BioRender. downward arrows (↓) indicate decreased expression, activity, or downregulation.. Created in BioRender. Carpinteyro, S. (2026) https://BioRender.com/jntugks (accessed on 23 March 2026).

The gut-brain axis is a dynamic bidirectional communication network that integrates neuronal, endocrine, immunological, and metabolic signals between the gastrointestinal tract and the CNS [106]. This intricate regulatory mechanism is crucial for neuronal excitability, synaptic plasticity, and metabolic homeostasis, all of which are significantly disrupted in epilepsy and obesity [107]. Recent research indicates that gut dysbiosis, characterized by an imbalance in the composition and function of the intestinal microbiota, serves as a significant factor in the pathogenesis of epilepsy and MetS [108,109] (Figure 4). Patients with epilepsy often demonstrate diminished microbial diversity, marked by a reduction in advantageous genera like *Bifidobacterium* and *Lactobacillus*, alongside an increased prevalence of pro-inflammatory taxa such as Proteobacteria and Firmicutes [109,110]. This microbial dysbiosis induces systemic inflammation and compromises intestinal barrier integrity, permitting bacterial endotoxins like lipopolysaccharide (LPS) to enter the bloodstream [111,112]. Circulating LPS triggers Toll-like receptor 4 (TLR4) signaling in microglia and astrocytes, leading to the synthesis of pro-inflammatory cytokines such as TNF-α, IL-1β, and IL-6, which increase neuronal excitability and heighten seizure susceptibility [113].

Gut-derived metabolites, particularly short-chain fatty acids (SCFAs) like butyrate, propionate, and acetate, serve as critical neuromodulators that influence brain function through epigenetic and metabolic pathways [114]. Under physiological conditions, SCFAs function as histone deacetylases (HDACs) inhibitors, promoting the expression of neuroprotective genes and anti-inflammatory pathways [115,116]. In cases of obesity and epilepsy, SCFA synthesis is significantly diminished. This curtailment leads to reduced activation of G-protein-coupled receptors (GPR41 and GPR43), impaired gut-brain communication, and attenuated neural resilience [117]. Furthermore, mitochondrial dysfunction bridges gut-brain interactions during metabolic and neurological stress [118]. Disrupted SCFA signaling impairs mitochondrial biogenesis by altering PGC-1α (peroxisome proliferator-activated receptor gamma coactivator-1α) activity, which results in decreased oxidative phosphorylation and elevated production of ROS [119,120]. The increase in ROS in neurons and glial cells induces oxidative stress, lipid peroxidation, and DNA damage, hence increasing neuronal hyperexcitability and facilitating seizure genesis [121]. The vagus nerve functions as an essential pathway for gut-brain communication, relaying microbial and hormonal signals to the brainstem and hypothalamus [122,123]. In cases of obesity and epilepsy, vagal tone is generally reduced, impairing parasympathetic control and the anti-inflammatory cholinergic system [124]. This dysautonomia enhances activation of the hypothalamic-pituitary-adrenal (HPA) axis and cortisol secretion, modifying glucose metabolism and neuronal excitability [125]. Chronic dysregulation of the HPA axis exacerbates visceral fat accumulation, insulin resistance, and cytokine release, thus creating a detrimental neuroendocrine feedback loop between obesity and epileptogenesis [126].

The tryptophan kynurenine pathway is a crucial metabolic connection between gut and brain function [127]. In dysbiosis and inflammatory states, intestinal and immunological cells excessively produce indoleamine-2,3-dioxygenase (IDO), redirecting tryptophan metabolism from serotonin synthesis to kynurenine biosynthesis [128]. Neuroactive metabolites, including quinolinic acid and kynurenic acid, traverse the BBB and influence glutamatergic transmission [129]. Increased quinolinic acid functions as an NMDA receptor agonist, facilitating excitotoxicity and seizures, while diminished serotonin levels lead to depressive and cognitive symptoms associated with epilepsy and obesity [130,131]. Systemic inflammation and endotoxemia resulting from dysbiosis further undermine BBB integrity. LPS-induced TLR4 activation in endothelial cells diminishes tight junction proteins, including claudin-5 and occludin, facilitating immune cell infiltration into the CNS [132,133]. This infiltration enhances microglial activation and neuroinflammatory signaling, sustaining epileptogenic circuits [13]. Endocrine hormones released by gastrointestinal and adipose tissues, including ghrelin, leptin, and adiponectin, regulate neuronal excitability within this axis [92,134]. Leptin, which typically amplifies GABAergic inhibition and curtails appetite, loses efficacy in the context of leptin resistance, a characteristic of obesity [89]. Hyperleptinemia accompanied by central leptin insensitivity impairs hypothalamic signaling, diminishes seizure threshold, and promotes weight gain [88]. In contrast, adiponectin demonstrates anti-inflammatory and insulin-sensitizing properties through AMPK signaling, enhancing mitochondrial integrity and synaptic function [88,91]. Decreased adiponectin levels in obesity and epilepsy hinder AMPK activation, intensify oxidative stress, and increase neuronal susceptibility [86].

Gut microbiota also influence neurotransmitter synthesis and metabolism, thereby directly modulating neural excitability [109]. *Lactobacillus* and *Bifidobacterium* species synthesise GABA, the principal inhibitory neurotransmitter, while other species modulate the turnover of serotonin, dopamine, and glutamate [135]. Dysbiosis shifts this balance toward excitatory dominance, reducing GABA synthesis and elevating glutamatergic activity [136]. Additionally, microbial metabolites regulate the expression of brain-derived neurotrophic factor (BDNF), a key mediator of synaptic plasticity that is frequently reduced in both obesity and epilepsy [137].

Recent research suggests that ketogenic diet therapy (KDT), commonly employed for drug-resistant epilepsy, partially achieves its effectiveness by modulating the gut microbiota [138]. KDT enhances the prevalence of *Akkermansia muciniphila* and *Parabacteroides* species, which generate compounds that fortify the intestinal barrier and mitigate inflammation [138]. These modifications enhance hippocampal GABA/glutamate ratios, hence enhancing seizure management [139]. Nonetheless, prolonged ketogenic diets may cause metabolic changes, highlighting the necessity for precise nutritional methods that balance antiseizure advantages with metabolic well-being [140]. Notwithstanding the mechanistic plausibility of the gut-brain axis in epilepsy and obesity, several critical limitations temper the current evidence. First, the vast majority of microbiome studies in epilepsy are cross-sectional in design, precluding causal inference; it remains unresolved whether gut dysbiosis precedes and contributes to seizure onset, or whether it develops as a consequence of recurrent seizures and antiseizure medication use. Second, although KDT demonstrably modulates gut microbiota composition and has established efficacy in drug-resistant epilepsy, its long-term cardiometabolic effects including dyslipidemia and elevated LDL-cholesterol must be carefully weighed in patients with pre-existing metabolic syndrome [140]. Third, SCFA supplementation studies, while promising in rodent seizure models, lack robust randomized controlled trial data in human epilepsy populations. These findings illustrate that the gut-brain axis operates as a bidirectional molecular system in which metabolic and neurological abnormalities mutually reinforce each other in epilepsy and obesity [141]. Gut dysbiosis, microbial metabolite imbalance, BBB disruption, neuroinflammation, mitochondrial dysfunction, and hormonal resistance form an interrelated pathophysiological network that propels both epileptogenesis and metabolic dysregulation [74]. Targeting these pathways by microbiome-based interventions, anti-inflammatory medications, and metabolic modulators may provide a unique therapeutic approach that concurrently enhances neurological and metabolic outcomes in patients with epilepsy and obesity [63,142].

## 7. Conclusions and Perspectives

**Figure 5:**
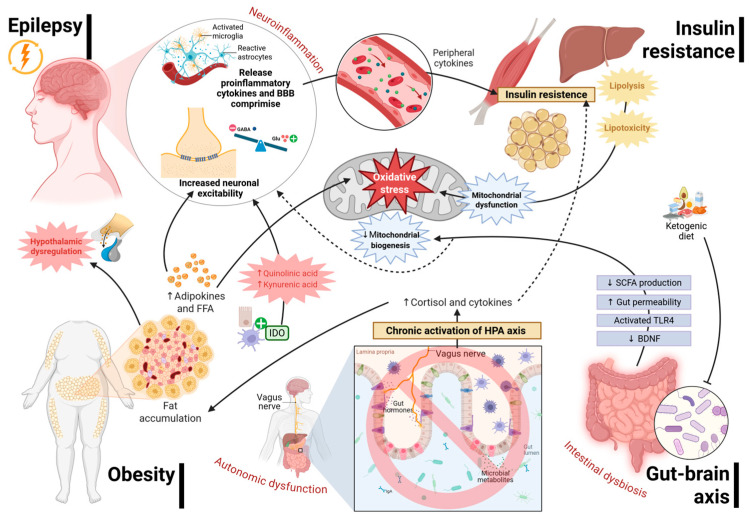
**Integrated neuro-metabolic model of the bidirectional relationship between epilepsy, obesity, and metabolic syndrome.** The figure illustrates six interconnected pathological domains—epilepsy, neuroinflammation, insulin resistance, intestinal dysbiosis/gut-brain axis disruption, obesity, and autonomic dysfunction—converging on a central hub of oxidative stress and mitochondrial dysfunction. Bidirectional arrows denote mutually reinforcing pathogenic relationships. Epilepsy drives microglial and astrocytic activation, releasing pro-inflammatory cytokines and compromising BBB integrity, which heightens neuronal excitability via glutamatergic upregulation and GABAergic suppression. Obesity promotes fat accumulation, adipokine and free fatty acid (FFA) dysregulation, and hypothalamic dysfunction, while peripheral insulin resistance induces lipolysis and lipotoxicity. Gut dysbiosis reduces short-chain fatty acid (SCFA) production, increases intestinal permeability, activates TLR4 signaling, and suppresses BDNF expression. Chronic activation of the hypothalamic-pituitary-adrenal (HPA) axis via the vagus nerve sustains cortisol and cytokine elevation, amplifying autonomic dysfunction. Indoleamine 2,3-dioxygenase (IDO) activation redirects tryptophan metabolism toward quinolinic and kynurenic acid, further modulating neuronal excitability. The ketogenic diet is depicted as a therapeutic modulator of the gut-brain axis. Collectively, these pathways establish a self-perpetuating neuro-metabolic continuum in which neurological and systemic metabolic dysfunction are mechanistically inseparable. Created in BioRender. Carpinteyro, S. (2026) https://BioRender.com/h412d3k (accessed on 23 March 2026).

The reciprocal relationship between epilepsy, obesity, and metabolic syndrome represents a paradigm shift in understanding neurological disorders as systemic conditions with profound metabolic foundations. Mounting evidence suggests that neuronal hyperexcitability, mitochondrial dysfunction, oxidative stress, and chronic inflammation are not confined to the central nervous system (CNS) but are inextricably linked to peripheral metabolic disturbances. This shared pathophysiology creates a self-perpetuating cycle where neuroinflammation and insulin resistance reinforce both neuronal vulnerability and systemic metabolic dysfunction. From this perspective, it is redefined: no longer viewed solely as a state of abnormal electrical activity it is recognized as a multifaceted neuro-metabolic disorder emerging from the intersection of cellular, endocrine, and immunological dysfunctions. At the molecular level, shared mediators such as cytokines, adipokines, and ROS constitute a convergent biochemical substrate that couples neural excitability to metabolic stress, as illustrated in the integrated model presented in Figure 5. Adipokine dysregulation, particularly leptin resistance and hypoadiponectinemia, alters signaling in the hypothalamus and hippocampus, precipitating excitotoxic cascades and cognitive deficits. Cerebral insulin resistance similarly disrupts synaptic plasticity, neurotransmitter homeostasis, and mitochondrial energetics, resulting in a metabolic milieu that lowers the seizure threshold. Processes are further compounded by dysregulation of the gut-brain axis, wherein intestinal dysbiosis and microbiota-derived metabolites increase enhance BBB permeability, activate microglia, and drive systemic inflammation.

Collectively, these findings underscore the need to conceptualize epilepsy and metabolic disorders as interconnected components of a unified neuro-metabolic continuum. This conceptual framework carries important therapeutic implications from a translational perspective. First, it supports the integration of metabolic profiling and gut microbiome assessment into the routine evaluation of individuals with epilepsy, particularly those with obesity or metabolic syndrome. Early identification of metabolic imbalances may enable the development of personalized therapeutic strategies aimed at restoring systemic and neural homeostasis. Second, interventions targeting shared molecular such as oxidative stress, cellular dysfunction, and inflammatory signaling may provide dual benefits by improving both seizure control and metabolic regulation. Pharmacological interventions including AMPK activators, PPAR-γ agonists, and insulin sensitizers alongside nutraceuticals with potent antioxidant and anti-inflammatory properties, represent promising adjunctive therapies to conventional antiseizure medications (ASMs). Moreover, modulating the gut microbiota through probiotics, prebiotics, or meticulously formulated diets, e.g., ketogenic or Mediterranean diets, offers a viable strategy for restoring gut-brain homeostasis. Such interventions aim to seizure outcomes while simultaneously mitigating, rather than exacerbating metabolic comorbidities. Future research should prioritize longitudinal and mechanistic studies aimed at elucidating the temporal dynamics of neuro-metabolic interactions, with particular focus on the roles of systemic inflammation, cellular bioenergetics, and gut-derived metabolites in epileptogenesis. The integration of multi-omics approaches, including metabolomics, transcriptomics, and metagenomic sequencing will be essential for identifying molecular signatures predictive of metabolic risk and seizure susceptibility. Moreover, advanced neuroimaging and metabolic mapping techniques may elucidate how peripheral insulin resistance and lipid dysregulation alter neural network connectivity and excitability in vivo. These findings deepen our mechanistic insight and support the development of integrative therapeutic frameworks transcend the traditional boundaries of neurology and endocrinology. Collectively, the available evidence substantiates the hypothesis that epilepsy, obesity, and metabolic syndrome are interconnected through shared cellular and molecular pathways, involving neuroinflammation, oxidative stress, adipokine dysregulation, insulin resistance, and disruption of the gut–brain axis. Viewing epilepsy as a systemic neuro-metabolic condition reframes its therapy, moving beyond a solely neurocentric approach to one that prioritizes metabolic equilibrium and inter-organ communication. This comprehensive perspective not only deepens our understanding of epileptogenesis but also heralds a new era of personalized, metabolism-focused neurology. Such an approach can more effectively address the intricate molecular interactions underpinning both seizures and metabolic disorders.

## Figures and Tables

**Table 1:** Adipokine dysregulation in obesity and its cellular and molecular involvement in epilepsy.

Adipokine	Status and Function in Obesity	Cellular and Molecular Role in Epilepsy	Key References
Leptin	**Increased** (leptin resistance). Hyperleptinemia is common in obese individuals and in PWE treated with valproate. Leptin regulates energy homeostasis, appetite, and reproductive function via hypothalamic Ob-R signaling.	Leptin modulates neuronal excitability through its Ob-R receptor, regulating neurotransmitter release and synaptic plasticity. Leptin resistance in the hypothalamus contributes to increased appetite, metabolic dysfunction, and reduced GABAergic inhibition, promoting neuronal hyperexcitability.	[33,34,35,88,89]
Adiponectin	**Decreased**. Its reduction is associated with insulin resistance and metabolic syndrome. Adiponectin exerts anti-inflammatory and insulin-sensitizing effects via AdipoR1/R2 receptors.	Adiponectin exerts neuroprotective and anti-inflammatory effects by activating the AMPK pathway. Decreased adiponectin reduces mitochondrial biogenesis and neuronal antioxidant capacity, increasing oxidative stress and vulnerability to epileptic seizures.	[36,41,88,90,91]
Resistin	**Increased**. Secreted by adipocytes and macrophages, it promotes insulin resistance and the release of pro-inflammatory cytokines via NF-κB activation.	Promotes microglial activation and the expression of TNF-α and IL-6 in the brain, amplifying neuroinflammation and synaptic dysfunction. Contributes to the alteration of neuronal ion channels and the reduction in GABAergic inhibition.	[13,18,83]
Ghrelin/NeuropeptideY (NPY)	**Dysregulated.** Alteration of the ghrelin–NPY axis, which regulates appetite and energy metabolism. Ghrelin is typically decreased in obesity; NPY tone is chronically elevated.	Ghrelin has neuroprotective and anticonvulsant effects via GHS-R1a signaling. NPY imbalance disrupts the excitatory–inhibitory balance in the hippocampus. Dysfunction of this axis contributes to metabolic vulnerability and a higher frequency of seizures.	[92,93]
Visfatin (NAMPT)	**Elevated** in obesity; acts as a pro-inflammatory mediator and modulator of energy metabolism through NAD^+^ biosynthesis. Also functions as an extracellular cytokine-like molecule.	Regulates NAD^+^-dependent mitochondrial function and can induce neuronal oxidative stress. High levels are associated with chronic neuroinflammation and alterations in cortical excitability.	[83,84,85]
Apelin	**Increased** in obesity; modulates insulin sensitivity, angiogenesis, and cardiovascular function via the APJ receptor.	In the brain, modulates neuronal excitability and neurogenesis. Its dysregulation contributes to oxidative stress and metabolic impairment in epilepsy.	[83,84]
Omentin	**Decreased**; its reduction is associated with chronic inflammation and insulin resistance. Under physiological conditions it exerts anti-inflammatory and insulin-sensitizing effects.	Has anti-inflammatory properties and improves insulin signaling. Reduced omentin in epilepsy exacerbates oxidative stress and mitochondrial dysfunction.	[66,67,83]
Adipsin	**Altered**; involved in the regulation of complement activation and lipid metabolism. Its expression is typically reduced in established obesity due to adipocyte dysfunction.	Its dysfunction is linked to increased neuronal damage induced by inflammation and oxidative stress.	[5,18,67]

Table 1 summarizes the principal adipokines whose expression or signaling is altered under conditions of obesity and metabolic syndrome, detailing their functional status, their established or proposed mechanisms of action in the central nervous system, and the key references supporting each entry. Adipokines are listed in order of mechanistic relevance to epileptogenesis as discussed in the text. Abbreviations: AMPK, AMP-activated protein kinase; GABA, γ-aminobutyric acid; GHS-R1a, growth hormone secretagogue receptor type 1a; NAD^+^, nicotinamide adenine dinucleotide; NAMPT, nicotinamide phosphoribosyltransferase; NF-κB, nuclear factor kappa-light-chain-enhancer of activated B cells; NPY, neuropeptide Y; Ob-R, leptin receptor; TNF-α, tumor necrosis factor-alpha; IL-6, interleukin-6; Adipo R1/R2, adiponectin receptor 1/adiponectin receptor 2; APJ, apelin receptor.

## Data Availability

No new data were created or analyzed in this study. Data sharing is not applicable.

## Abbreviations

AMPK	AMP-Activated Protein Kinase
ASM	Antiseizure Medication
BBB	Blood–Brain Barrier
BDNF	Brain-Derived Neurotrophic Factor
CNS	Central Nervous System
ER	Endoplasmic Reticulum
FFA	Free Fatty Acids
GABA	γ-Aminobutyric Acid
GLUT	Glucose Transporter
GPR41/GPR43	G-Protein–Coupled Receptor 41/43
HDAC	Histone Deacetylase
HPA Axis	Hypothalamic–Pituitary–Adrenal Axis
IDO	Indoleamine 2,3-Dioxygenase
IL-1β	Interleukin-1 Beta
IL-6	Interleukin-6
IRS	Insulin Receptor Substrate
KDT	Ketogenic Diet Therapy
LPS	Lipopolysaccharide
MetS	Metabolic Syndrome
NMDA	N-Methyl-D-Aspartate Receptor
NPY	Neuropeptide Y
PGC-1α	Peroxisome Proliferator–Activated Receptor Gamma Coactivator-1 Alpha
PPAR-γ	Peroxisome Proliferator–Activated Receptor Gamma
PWE	Patients With Epilepsy
ROS	Reactive Oxygen Species
SCFA	Short-Chain Fatty Acid
TLR4	Toll-Like Receptor 4
TNF-α	Tumor Necrosis Factor Alpha
UPR	Unfolded Protein Response
VPA	Valproic Acid
